# Supplementary material for: Complexome profiling of the Chlamydomonas psb28 mutant reveals TEF5 as an early PSII assembly factor
Source: Plant Cell. 2025 Mar 17;37(6):koaf055. doi: 10.1093/plcell/koaf055 (PMC12164590; doi:10.1093/plcell/koaf055)
Supplement: koaf055_Supplementary_Data [file koaf055_supplementary_data.zip › psb28_interactive.zip!/plots/intensity/qualityPlots/peptides/MPC5_Cre03.g172300.t1.2.html]

### Cre03.g172300.t1.2

Trivial name: MPC5  
SeqCoverage [median]: 36.20  
Mass: 37.44 kDa  
  
 

| Sequence | MissedCleavage | Mass [Da] | wtIntensity | muIntensity | Score |
| --- | --- | --- | --- | --- | --- |
| FGAFENTVQAIYK | 0 | 1486.75 | 94197.87 | 115432.86 | 279.77 |
| FGLYEYFK | 0 | 1065.52 | 353.00 | 0.00 | 70.00 |
| FGLYEYFKK | 1 | 1193.61 | 1872.35 | 27605.24 | 91.32 |
| FVAQEGWGGLFK | 0 | 1337.68 | 11068.15 | 0.00 | 134.68 |
| GATVGDIVK | 0 | 858.48 | 22703.23 | 40207.02 | 113.89 |
| GISTGFR | 0 | 736.39 | 7477.55 | 20355.37 | 98.42 |
| GLSDGLPK | 0 | 785.43 | 73686.79 | 69936.25 | 130.04 |
| KTYADMAGEEVAK | 1 | 1411.67 | 0.00 | 898.26 | 40.48 |
| QIPYTMMK | 0 | 1010.49 | 13984.10 | 66990.50 | 69.42 |
| TYADMAGEEVAK | 0 | 1283.57 | 31260.30 | 84009.46 | 183.67 |
| TYADMAGEEVAKK | 1 | 1411.67 | 17593.89 | 19282.24 | 107.14 |
| VKVQTVPGFAK | 1 | 1172.69 | 8372.10 | 15628.00 | 57.88 |
| VLVAEQGAAGLFK | 0 | 1301.73 | 165404.49 | 90922.99 | 240.10 |
| VQTVPGFAK | 0 | 945.53 | 257269.00 | 301246.96 | 180.45 |
| VSMGLPTTGSVEEK | 0 | 1433.71 | 131836.16 | 156901.35 | 169.98 |
|  |  |  | 837079.0 | 1009416.5 |  |
